# Mitral Obstruction—Treatment

**Published:** 1873-12

**Authors:** Wm. Pepper


*MEDICAL AND SURGICAL REPORTER - December,* 1873:

Treatment of Mitral Obstruction—*By Wm. Pepper, M.D.*—In treating mitral obstruction, we must endeavor to preserve the tonicity of the muscular fibres of the heart, to prevent too rapid and excited contractions of the left ventricle, and to overcome the tendency to pulmonary embarrassment. Digitalis fulfills the first indication better than any other drug, and it is important for you to remember that not only in this disease, but whenever you find the cavities of the heart subjected to a continuous over-strain, digitalis is the remedy to be employed. The dose for an adult is ten drops of a good tincture, and for a child over ten years of age, five or six drops, repeated in each instance four times daily; it should be used steadily, and not merely as a palliative, when there is palpitation.

Quinia, strychnia and arsenic stand next to this medicine in their power of promoting healthy action of the heart, and, together with iron, may be used from time to time in addition to digitalis, as they all tend to improve nutrition and maintain the tone of the muscular system.

When the contractions of the left ventricle become frequent and excited, tincture of aconite, or fluid extract of veratrum viride, should be given, the proper dose of either of which, for an adult, is three drops three or four times a day, which may be cautiously increased. Belladonna may also be employed under these circumstances, either in the form of a plaster applied over the region of the heart, or administered internally.

Pulmonary congestion is prevented by keeping up the circulation of the skin by sufficient clothing and moderate exercise. The diet should be plain and unstimulating, and care should be taken to avoid all excitement and over-fatigue, as well as undue exposure to atmospheric changes. Finally, if torpor of the liver, or any functional disturbance of the abdominal organs is present, it must be immediately remedied.

If the lungs have become engorged, we may use, according to the condition of the patient, wet or dry cups, with alkaline and stimulating expectorants, such as muriate of ammonia, carbonate of potassa, squills, etc. When there is hæmoptysis, the combined employment of ergot and digitalis will be followed by marked relief.

